# Unleashing the Power of Reliance for Post-Approval Changes: A Journey with 48 National Regulatory Authorities

**DOI:** 10.1007/s43441-024-00677-8

**Published:** 2024-07-24

**Authors:** Francesca Mangia, Yameng (Melly) Lin, John Armando, Kareny Dominguez, Vera Rozhnova, Susanne Ausborn

**Affiliations:** 1 F.Hoffmann-La Roche, Grenzacherstrasse 124, Basel, 4058 Switzerland; 2 Genentech, Inc., 1 DNA Way, South San Francisco, CA 94080 USA

**Keywords:** Reliance, Post-Approval Changes, Convergence, Transparency

## Abstract

Post-approval changes (PACs) to marketed products are routinely introduced to continuously enhance the product lifecycle management. However, bringing a chemistry, manufacturing and control (CMC) change through the global health authorities can be a complex and lengthy process taking up to several years, therefore negatively impacting supply continuity. In order to accelerate the review and approval of regulatory submissions and ensure continuous supply to patients, the World Health Organization (WHO) is strongly supporting the implementation of reliance among National Regulatory Authorities (NRAs). While some promising developments have been made with the use of reliance pathways for initial marketing authorizations, reliance is still not widely used for PACs. With the support of the European Medicines Agency (EMA) and WHO, Roche launched a reliance pilot based on EMA approval to file a supply critical variation for a monoclonal antibody. The variation constitutes major changes to the approved manufacturing process. Sameness of the product is ensured by submitting to all participants the same variation package as in the EU. The objectives of the pilot are to ensure continuous supply of this critical medicine by targeting global approval in 6.5 months, to promote regulatory convergence by waiving country specific requirements, and enhance greater transparency by sharing EMA Committee for Medicinal Products for Human Use (CHMP) final assessment report and Q&As to participating NRAs. Globally 48 NRAs have agreed to join the pilot. This article outlines the process of establishing the pilot project, including a planning phase and an engagement phase with the EMA, WHO and the participating NRAs.

## Introduction

Post-approval changes (PACs) are crucial for the continuous supply of essential medicines, responding to demand surges, change in regulatory requirements, and technological advancements. Accelerated development products, especially those via expedited pathways, often require many post-approval CMC changes to be implemented timely. Globally harmonizing these changes is challenging due to varying national regulations and approval processes, which can impact drug availability [1]. Regulatory submissions, dominated by PACs, are vital for maintaining supply and improving existing processes [2].

International efforts aim to streamline lifecycle management, with the WHO publishing variation guidelines for biotherapeutics [3] and vaccines [4], offering clear risk-based reporting and data requirements. The WHO’s Good Reliance Practice (GRelP) Guideline [5] encourages regulatory collaboration to expedite access to quality health products. The International Conference of Drug Regulatory Authorities (ICDRA) and WHO recommended NRAs to adopt reliance throughout product lifecycles [6]. International Pharmaceutical Regulators Programme (IPRP) have also developed a Q&A document [7] on key aspects for reliance.

The Covid-19 pandemic catalyzed regulatory collaboration, with initiatives like the International Coalition of Medicines Regulatory Authorities’ (ICMRA) development of a Quality Knowledge Management System and two reliance pilots in 2021, including one for CMC changes and another for joint inspections. These pilots aim to create frameworks for collaborative regulatory assessments and inspections.

Industry associations contribute through position papers [8, 9] tackling common challenges and promoting reliance pathways. Sanofi’s recent PAC reliance pilot tested GRelP principles, gaining insights into the practicalities of reliance [10].

To bring reliance into action and thereby supporting the efficient global implementation of reliance, Roche launched a PAC reliance pilot for major process changes of a monoclonal antibody. This article outlines the pilot’s initiation and the initial lessons learned.

## Methods

### Case Study Selection Criteria

The Roche PAC reliance pilot was selected based on the following criteria.

1. The medicinal product is used for treating life-threatening diseases.
2. It involves a major supply critical variation with high public health and business impact.
3. The reference country’s approval is expected in 6 to 12 months, which allows sufficient time to engage stakeholders and NRAs around the world.
4. Assessment report from reference agency is/will be available.
5. There is no negative impact on the overall product strategy based on the considerations for change implementation, grace period policies and acceptance of parallel submissions in the participating countries, impact of accelerated approval on the supply planning, etc. Grace period refers to the period of time between the approval of the change and the importation of post-change material. During the grace period, the pre-change material can still be imported and used. Parallel submission refers to the possibility to submit a new filing (e.g. PAC) while the review of a previous filing is still ongoing.

Based on the above criteria, a major drug substance manufacturing process change for a monoclonal antibody was selected which represents a complex and critical variation aimed at ensuring long-term supply resiliency. The change was submitted as a Type II variation to EMA and the approval was received in 4 months.

### Criteria for the Selection of Countries

This major variation impacted 129 countries around the world, 84 of them were included in this pilot. The other countries were excluded from the pilot for the following reasons: (1) 30 EEA countries covered by EMA’s approval; (2) Countries which are typically reference agencies; (3) Countries with unpredictable intellectual property management preventing sharing of the same EU dossier; (4) The proposed variation is not required to submit according to the local legislation; (5) Low business priority (6) Complex local requirements including registration testing.

### Regulatory Landscape for the Variation in the Scope

According to IFPMA/EFPIA/Vaccine Europe, the current global regulatory landscape for PACs is fragmented [11]:

- Inconsistent classification systems.
- Specific and supplementary local data and format requirements.
- Unpredictable and variable approval timelines.
- Divergent interpretation and decisions by regulators based on the same data.
- Variable implementation periods after completed regulatory action.

The above challenges also existed for the global filing of the change selected for this pilot.

### Pilot Description and Objective

In this PAC reliance pilot, participating NRAs have the opportunity to apply reliance by leveraging the EMA assessment and approval to reduce the review timelines. The participating NRAs can decide, upon receipt of the EMA CHMP final assessment report and approval, if they would fully or partially rely on the EMA evaluation in reaching their own independent decision.

The objectives of the PAC reliance pilot include:

- Ensuring continuous supply of this critical medicine to patients by targeting the approval timeline in 6.5 months instead of 2.5 years.
- Promoting regulatory convergence by reducing country specific requirements, e.g. local testing and country specific documents and aligning the submission package to the documentation required by EMA.
- Enhancing greater transparency to NRAs by sharing EMA CHMP final assessment report and Q&As.

**Fig. 1:**
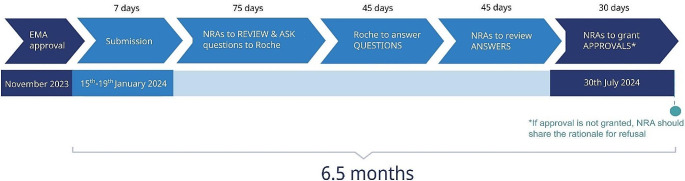
Pilot stages and their respective timelines: 7 days for submission of the dossier, 75 days allocated for the NRAs to review and raise questions, 45 days for Roche to respond, which is followed by 45 days for the NRAs to review Roche’s responses and finally grant approval within another 30 days. This leads to a total and maximum time of 6.5 months from the time of submission to approval. *Created using Inkscape*

To participate in the pilot, the following prerequisites needed to be agreed by NRAs:

- Adhere to the proposed approval timeline (max 6.5 months, Fig. 1) which is consistent with timeline suggested for major changes (3–6 months) in the WHO Guideline on procedures and data requirements for changes to approved biotherapeutic products (WHO, 2018).
- Accept EMA as a reference agency and follow EMA submission standards, i.e. EU variation guideline, ICH Q5E and WHO principles by avoiding requesting country specific documents or data.
- Waive sample testing by relying on Certificates of Analysis (CoAs) issued by the manufacturer of the product that was approved and routinely inspected by EMA.

### Overall Process and Timeline

**Fig. 2:**
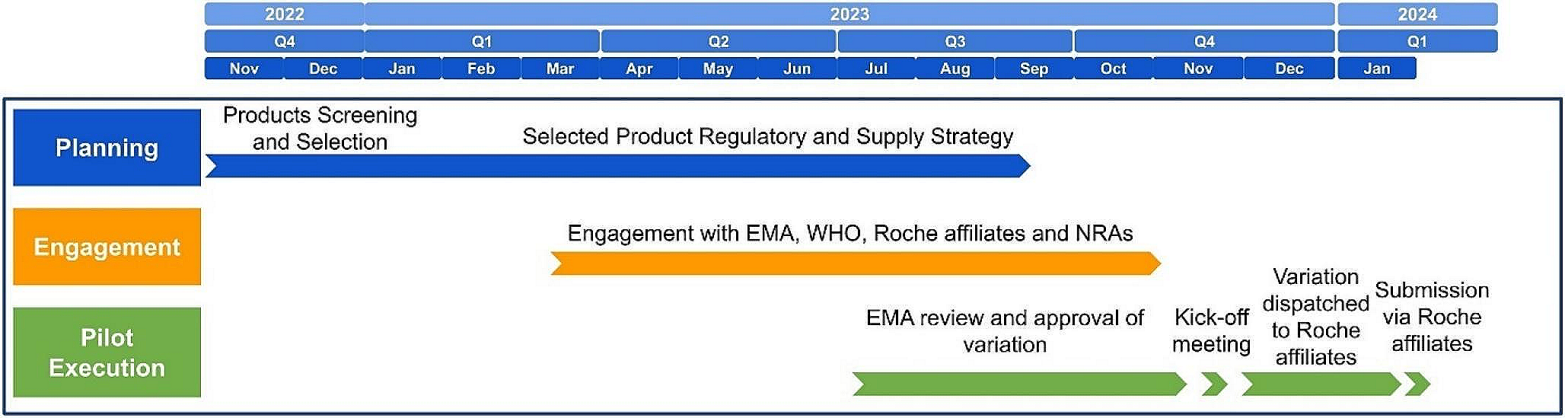
General process and timeline of PAC reliance pilot. *Created using Inkscape*

The PAC reliance pilot involves three phases, which are outlined in Fig. 2.

- **Planning phase**: Project screening criteria were defined as described in the method section and the product was selected. The regulatory and supply strategy was defined for each country based on supply critical factors.
- **Engagement phase**: During this phase, the company approached EMA, WHO and NRAs impacted by this variation.
- **Execution phase**: After EMA’s approval of the variation, the company embarked on the pilot Execution phase which includes a kick-off meeting, pilot submission to the participating NRAs, review, Q&A and approval.

### Success Factors

#### Support from Key Stakeholders (EMA, WHO)

EMA is a leading example for transparency among regulatory agencies being as open as possible about its review process and how regulatory decisions were made. Both EMA and WHO are strong advocates for broader implementation of reliance pathways across the globe and EMA has a long track record as a leading reference agency in the WHO Collaborative Registration Procedure. EMA and WHO’s support of the pilot and encouragement for NRAs to leverage the EMA final assessment report and Q&As to reach a national decision were considered critical factors for the high participation rate of NRAs in the pilot. Furthermore, EMA’s openness to provide opportunities to clarify NRA questions regarding the assessment documents helped foster an open, collaborative and transparent pilot process.

#### Product Sameness

According to WHO, assuring sameness of product is essential for the use of reliance (WHO, 2021). With this post-approval change, end-to-end updated drug substance manufacturing process will be introduced for all countries where the product is registered. The same drug substance manufacturing site, drug product manufacturing process, site, formulation, and container closure system will be used for global supply. Therefore the same material will be supplied to all the countries in the world. Furthermore the same variation dossier, as for EMA, was submitted to all participating NRAs.

#### Transparency

To ensure full transparency, the participating NRAs had access to the EMA CHMP final assessment report (with personal data redactions to ensures compliance with the EU legislation on the protection of personal data, according to the General Data Protection Regulation (GDPR, 2016), EMA’s questions and company’s responses which were included in the national submission package. In addition, during the review period the company will provide a single Q&A document, which will include all the questions received from participating NRAs and company’s responses. This single Q&A document will be shared with all participating NRAs, as well as with WHO and EMA. In order to facilitate sharing of questions and responses in real time, a cloud based Q&A platform developed by a 3rd party is being used.

## Results

### Planning Phase

The pilot planning phase started with defining pilot selection criteria. After successful product selection a cross-functional PAC Reliance Pilot team (referred to as ‘the team’) was formed with representatives from the CMC International Operations department and CMC Regulatory Policy to develop a detailed pilot roadmap and define internal and external engagement plans (Fig. 2).

The team firstly made the decision to use EMA as the reference country considering EMA’s strong commitment and support to international collaboration and reliance and availability of the final CHMP assessment report. WHO was considered as a key stakeholder to engage considering its crucial role in driving and supporting the implementation of regulatory reliance globally.

Besides external engagement activities, a detailed global filing plan was established to assess the impact on internal resources and drug supply with the consideration of submission and approval timeline of related PACs, possibility of parallel submission, global implementation timeline, grace period, etc.

### Engagement Phase

#### First Engagement Meeting with EMA & WHO

The company held the first engagement meeting with EMA and WHO in March 2023 to present objectives, plans and timelines on the PAC Reliance Pilot. Both stakeholders were very positive and supportive of Roche efforts to initiate the pilot using reliance to get timely approval of an important variation, as it is also in line with EMA’s commitment to prioritize high quality, robust and rapid assessment of medicines in oncology.

#### Engagement with NRAs

From June 2023 to October 2023, Roche affiliates, with the support from the team, contacted NRAs in 84 countries to present the pilot by email, tele/video conference or Face-to-Face meeting. An introduction package was submitted to all NRAs, including a slide deck on PAC reliance pilot introduction, a Q&A document outlining the background and addressing critical aspects of the pilot, the rationale for the change classification and an invitation letter from EMA to NRAs to practice reliance leveraging their assessment and participate in the pilot.

#### NRAs Participation

**Fig. 3:**
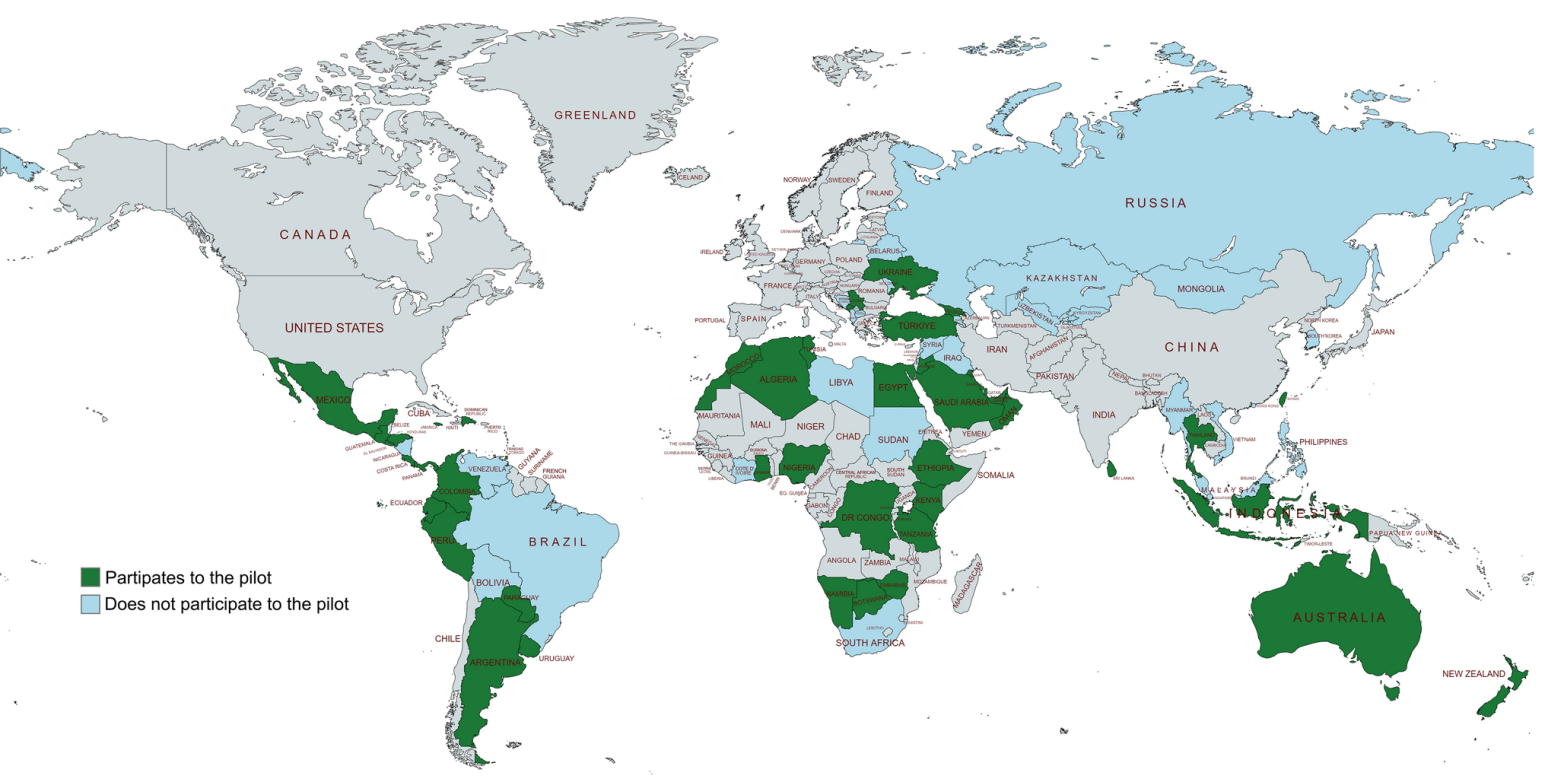
World map showing pilot participation status. In green are shown countries participating in the pilot, while in cyan are shown countries that did not participate. Created using mapchart.net

Among the group of 84 NRAs targeted for the pilot program, 48 (57,1%) agreed to participate, while 31 declined and 5 did not provide a definitive response. Figure 3 provides a visual representation of the global distribution of the participating NRAs in the pilot program.

**Fig. 4:**
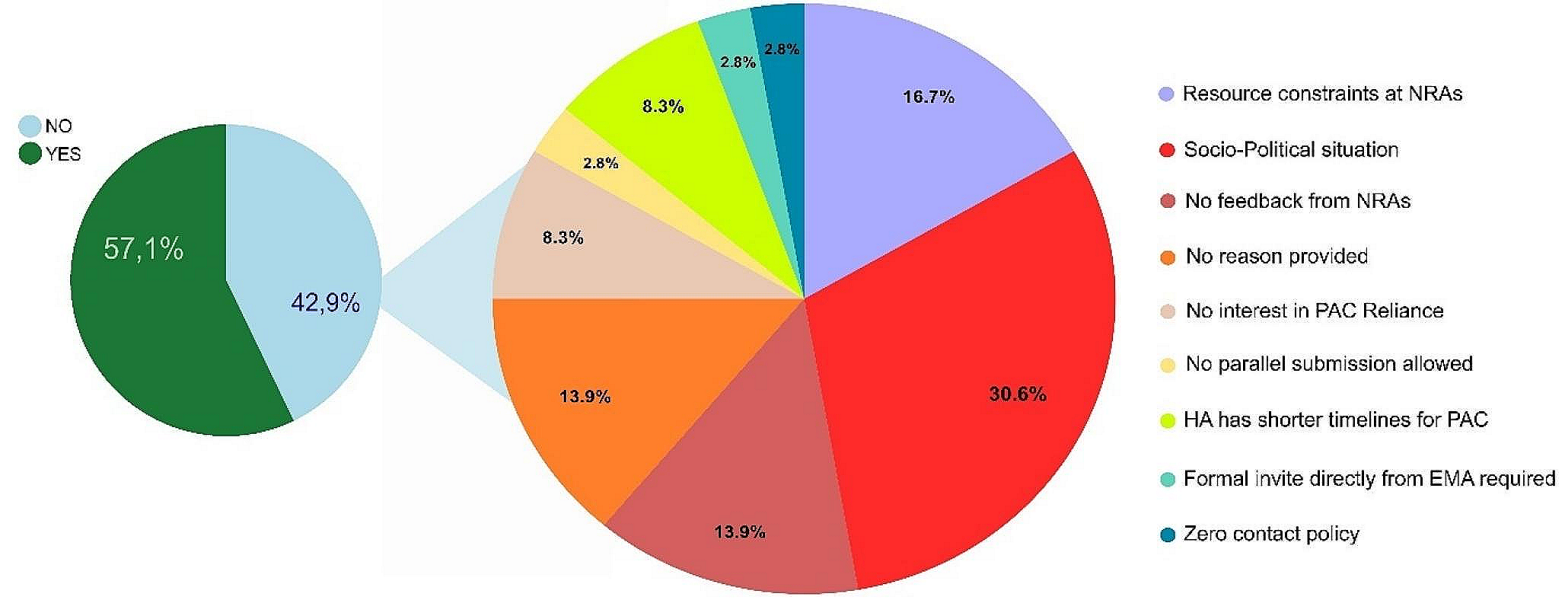
The pie chart on the left displays the percentage of NRAs participating in the pilot and those that declined. The pie chart on the right shows the reasons provided by the declining NRAs for not participating in the pilot. *Created using Inkscape*

One of the primary objectives of the pilot was to uncover any potential obstacles, such as regulatory or legislative challenges, that may hinder the successful implementation of reliance. The pie chart (Fig. 4) provides analysis of reasons for refusal to participate. Some countries, including Mongolia and Myanmar, declined the pilot because their standard approval timelines for such a change are shorter than the proposed pilot timeframe. Malaysia was not able to join the pilot due to the NRA portal limitations for parallel submission. In some countries, including Palestine, Lebanon, Libya, Syria and Venezuela, the current socio-political situation made their participation in the pilot not possible. South Korea preferred not to participate in the pilot as their current efforts are focused on collaborative review. Certain countries either did not respond within the specified timeframe (Aruba, Brazil, Mauritius) or encountered resource constraints within their respective NRAs (Albania, Bolivia, North Macedonia, Iraq and South Africa). These insights provided valuable information about the barriers that need to be addressed in order to facilitate the implementation of reliance principles in the future.

### Execution Phase

#### Kick off Meeting with all Stakeholders and NRAs

Following EMA’s approval, a pilot kick off meeting was scheduled with EMA, WHO and all participating NRAs. The purpose of the meeting was to emphasize the benefits of reliance and introduce the pilot framework and guardrails including the objectives, timeline and Q&A handling. The participating NRAs engaged actively in the discussion with questions around assessment reports, review timeline, provision of country specific requirements, reliance pathway, etc.

#### Submission Achieved within One week

In order to allow the same review times for all participating NRAs, 48 Roche affiliates and agents submitted the dossier package simultaneously (within one week time frame) to their respective NRAs using the national submission procedures. This achievement is especially remarkable considering the submission restrictions in some markets, e.g. limitations in portal capability, payment procedure, appointment for submission, etc.

#### Convergence of PAC Requirements

According to the sponsor’s internal database, 38 NRAs have country specific requirements for the change (Table 1) in scope. At the time of submission, all NRAs accepted the same package as EMA without requesting any country specific documents, which demonstrates the willingness for NRAs to align regulatory requirements. In 5 countries this major process change may be considered as a new registration, however with early communication and further engagement meetings, all 5 NRAs agreed to align their reporting category with the reference country, EMA. For this specific pilot only one country, Egypt, requires local retesting for the first shipped batch after the variation is approved, and we requested a waiver of sample testing as part of the PAC Reliance pilots.

**Table 1:** Country-specific requirements for the variation application

Requirements	Country/ies
3.2.S.4.4 Batch Analyses - Chromatograms	Mexico
3.2.S.7.3 Stability Data - Chromatograms	
3.2.P.8.3 Stability Data - Chromatograms	
Batch Traceability - Manufacturing and Packaging Sheets	
Excipients Certificates of Analysis	
Packaging Materials Certificates of Analysis	
Declaration on corresponds in Specifications and Certificate of Analysis	
3.2.P.8.3 Stability Data - Addendum	Colombia, Mexico
Swissmedic approval letter	Algeria, Tunisia
Local testing	Egypt
Flowchart including manufacturing Licenses and GMP documents	Algeria
Commitment to provide stability data covering shelf life	Indonesia
Drug Substance Certificate of Analysis	Algeria, Argentina, Australia, Bahrain, Botswana, Brunei, Colombia, Costa Rica, Democratic, Republic of Congo, Ecuador Egypt, El Salvador, Ethiopia, Ghana, Guatemala, Indonesia, Israel, Jordan, Kenya, Kuwait, Mexico, Montenegro, Morocco, Namibia, New Zealand, Nigeria, Oman, Saudi Arabia, Serbia, Sri Lanka, Taiwan, Tanzania, Thailand, Tunisia, Turkey, Ukraine, Uruguay, Zimbabwe.
Drug Product Certificate of Analysis	Algeria, Argentina, Bahrain, Botswana, Brunei, Colombia, Costa Rica, Indonesia, Jordan, Mexico, Namibia, Saudi Arabia, Serbia, Ukraine, Zimbabwe.
Comparative Batch Analysis	Algeria, Argentina, Australia, Bahrain, Botswana, Brunei, Colombia, Costa Rica, Democratic Republic of Congo, El Salvador, Ethiopia, Ghana, Indonesia, Israel, Jordan, Kenya, Kuwait, Mexico, Montenegro, Morocco, Namibia, New Zealand, Nigeria, Oman, Saudi Arabia, Serbia, Sri Lanka, Taiwan, Tanzania, Thailand, Tunisia, Turkey, Ukraine, Zimbabwe.
Declaration of approved Product Table of Content	Peru
Declaration on absence of residual solvent testing	Colombia
Declaration on the start of the Drug Product stability study	Brunei, Mexico, Thailand.
Declaration specifying what is not changing	Argentina, Bahrain, Brunei, Mexico, Saudi Arabia, Thailand.
TWIMC letter that there are no modification to impurity profile of DS	Indonesia

During the pilot program, some participating NRAs made specific requests to the company in order to be part of the initiative. A crucial requirement for the pilot to proceed was to harmonise the filing requirements to the fullest extent by adopting a uniform approach. Hence, the company carefully evaluated the individual requests from each NRA to determine if minor modifications could be considered acceptable. Table 2 provides an overview of these requests and the company’s stance on each of them. As Argentina and Colombia requested the variation dossier in Spanish for their participation in the pilot, the company agreed to provide a translated Spanish dossier to all other countries requiring it, to accommodate their request and ensure fairness.

**Table 2:** Analysis of participating NRAs’ specific local requests and company’s position

Country	Request	Company’s position
Argentina, Colombia	Dossier Translation Request	Provided to all Latam countries requiring Spanish dossiers
Colombia	Pre-submission of administrative documents in July.	Provided to facilitate expedited process

#### Parallel Submission and Grace Period

Navigating the complexities of the global regulatory environment can be quite challenging, especially when handling multiple changes affecting the same product and facing variable and often unpredictable approval timelines.

The product in scope of the pilot was undergoing another major variation affecting the control strategy with hard implementation at the end of 2024. This control system change required parallel review with the manufacturing process change in several countries because of long approval timelines or the lack of grace period. Eight out of 48 NRAs that participate in the pilot do not allow the submission of a new PAC when the review of another PAC affecting the same CTD section is ongoing or no parallel filings are allowed at all. In line with the pilot objectives to harmonise the filing requirements, NRAs were asked to allow parallel submission.

6 NRAs granted parallel review in order to be able to participate in the PAC reliance pilot. Two NRAs didn’t accept parallel review but took other measures to accelerate the review of the ongoing PAC application so that it could be approved prior to the start of the pilot.

Another particular challenge arose from the fact that countries have different grace period policies for the implementation of the variation, that some countries do not even allow any grace period (i.e. after approval only post-change material can be supplied). Therefore customised regulatory and especially supply planning, was critical to ensure continuous supply to patients. Within the 48 NRAs, 5 NRAs usually do not permit any grace period. However, upon engagement with the health authorities, a minimum of 6 months grace period was granted to handle the complex supply situation.

## Discussion

Post-approval changes are critical to ensure uninterrupted supply and continuously improve existing medicines. However the high number of PACs introduced by companies paired with the current global regulatory complexity of PACs management poses a high risk of supply disruptions of existing medicines. Approaches that would enable a more efficient management of quality and supply improvements include regulatory convergence using science and risk-based approaches, adoption of international standards by regulatory authorities, clear guidelines with timelines, and risk-based classification and the use of reliance.

Encouraged by many positive developments towards reliance led by WHO, ICMRA and others, Roche has launched a PAC reliance pilot for a global filing using EMA as reference agency. The lessons learnt from the Pilot Planning Phase to the start of Execution Phase are summarised below:

1. Transparency and dialogue are crucial factors in establishing trust between industry and regulators. Sharing the same variation package as approved by EMA as well as the final CHMP assessment report and Q&A were key elements in this pilot. Worthy of consideration is that EMA is one of the few agencies which issues unredacted final CHMP assessment for PACs (Type IB and Type II), for which Sponsors only need to redact personal information of EU assessors to comply with the GDPR rules. As a result, EMA plays a significant role in facilitating the use of reliance around the world. By maintaining open communication, sharing relevant reliance documents and engaging with interested NRAs directly, we were able to establish the initial trust and confidence necessary for 48 NRAs to join the pilot program.
2. Equally important was the support provided from EMA and WHO, both strong advocates for reliance around the globe. The choice of the reference agency should be indeed dictated by its commitment in supporting reliance and the provision of the final CHMP assessment report, considering that European Public Assessment Report (EPARs) are not available for PACs. EMA’s participation in the pilot’s kick-off meeting, along with WHO, and the offer for clarification on questions related to the assessment report were influencing factors for the NRAs’ participation.
3. The product choice and supply impact were crucial for regulators’ agreement to join the pilot. The selected variation should be time-sensitive, supply impacting, and for a life-saving medicine. Additionally, it should impact a significant number of international markets to maximize its benefits.
4. The product strategy’s overall impact should be carefully assessed. This includes considerations such as global supply chain implications, grace period policies in affected countries, acceptance of parallel submissions of PACs, and the impact of accelerated approval on supply planning. etc. The divergent regulatory requirements add more complexity to the assessment and change implementation. The entire supply assessment exercises further reflect the needs for global harmonisation on the regulatory system for change management.
5. Affiliates are crucial in local interactions with NRAs and communicating about the pilot. However, engaging them can at times be challenging when proposing a new way of working outside the current regulatory framework. Close trustful collaboration among affiliates and the pilot team ensured consistent delivery of the pilot’s objective and reliance message to NRAs, ultimately securing their participation. Coordination with affiliates is essential for simultaneous submissions, especially in agent-managed countries.
6. The 84 impacted countries have diverse regulatory landscapes with differing change classification, dossier requirements and approval timelines. Some HAs find a 6.5-month approval time long, while for others, it is shorter than industry experience for post-approval changes which can take up to several years. The primary challenge is the unpredictability of PAC review and approval timelines due to varying change classifications and lack of procedural guidance potentially leading to supply challenges and therefore impact patients.

## Conclusion

The scale of this PAC reliance pilot is the largest ever conducted which demonstrates the strong interest and willingness of many NRAs across the globe in bringing reliance into practice for PACs. Transparency is key to building trust among industry and regulators. Applying reliance to the whole life-cycle of the product, including PACs, signifies a contribution towards global convergence and supports the ultimate goal of ensuring continuous supply for patients.

It is very encouraging to see that 48 countries have agreed to align their submission requirements with those of EMA, waiving, if any, additional national requirements and paving the way for a truly reliable pathway.

After the pilot completion, a second planned publication will provide insights on the Execution Phase, including details on the change review and approval by the 48 participating NRAs and their experience with the PAC Reliance pilot.

## Data Availability

No datasets were generated or analysed during the current study.
